# Metabolomics integrated genomics approach: Understanding multidrug resistance phenotype in MCF-7 breast cancer cells exposed to doxorubicin and ABCA1/EGFR/PI3k/PTEN crosstalk

**DOI:** 10.1016/j.toxrep.2024.101884

**Published:** 2024-12-25

**Authors:** Mai O. Kadry, Gamal Eldein Fathy Abd-Ellatef, Naglaa M. Ammar, Heba A. Hassan, Noha S. Hussein, Nahla N. Kamel, Maha M. Soltan, Rehab M. Abdel-Megeed, Abdel-Hamid Z. Abdel-Hamid

**Affiliations:** a National Research Center, Therapeutic Chemistry Department, Al Bohouth Street, Egypt; b National Research Center, Biology Unit, Central Laboratory for Pharmaceutical and drug industries Research Institute, Chemistry of Medicinal Plants Department, Al Bohouth Street, Dokki, Egypt.

**Keywords:** Breast cancer, ABCA1, Metabolomics, EGFR, PTEN

## Abstract

Resistance of cancer cells, especially breast cancer, to therapeutic medicines represents a major clinical obstacle that impedes the stages of treatment. Carcinoma cells that acquire resistance to therapeutic drugs can reprogram their own metabolic processes as a way to overcome the effectiveness of treatment and continue their reproduction processes. Despite the recent developments in medical research in the field of drug resistance, which showed some explanations for this phenomenon, the real explanation, along with the ability to precisely predict the possibility of its occurrence in breast cancer cells, still necessitates a deep consideration of the dynamics of the tumor's response to treatment. For this purpose the current study, combined both *in vitro* metabolomics and *in vivo* genomics analysis as the most advanced omics technologies that can provide a potential en route for inventing novel strategies to perform prospective, prognostic and diagnostic biomarkers for drug resistance phenomena in mammary cancer. Doxorubicin is the currently available breast cancer chemotherapeutic medication nevertheless; it was demonstrated to cause drug resistance, which impairs patient survival and prognosis by prompting proliferation, cell cycle progression, and preventing apoptosis, interactions between signaling pathways triggered drug resistance. In this research, *in vitro* metabolomics analysis based on GC-MS coupled with multivariable analysis was performed on MCF-7 and DOX resistant cell lines; MCF-7/adr cultured cells in addition to, further *in vivo* confirmation via inducing mammary cancer in rats via two doses of 7,12-dimethylbenz(a) anthracene (DMBA) (50 mg/kg and 25 mg/kg) proceeded by doxorubicin (5 mg/kg) treatment for one month. The metabolomics *in vitro* results pointed out that mannitol, myoinositol, glycine, α-linolenic acid, oleic acid and stearic acid have AUC values: 0.14, 0.5, 0.7, 0.1, 0.02, −0.02 (1, 1) respectively. Glycine and myoinositol metabolites provided the best discriminative power in the wild and resistance MCF-7 phenotypes. Meanwhile, *in vivo* results revealed a significant crosstalk between the alternation in oxidative stress biomarkers as well as Arginase II tumor biomarker and the molecular assessment of ABCA1 and P53 gene expression that displayed a marked reduction in addition to, the obvious elevation in resistance and apoptotic biomarkers EGFR/PI3k/AKT/PTEN signaling pathway upon DMBA administration. Data revealed a significant alternation in signaling pathways related to resistance upon doxorubicin administration that affect lipid metabolism in breast cancer. In conclusion, Metabolomics integrated genomics analysis may be promising in understanding multidrug resistance phenotype in MCF-7 breast cancer cells exposed to doxorubicin through modulating ABCA1/EGFR/P53/PI3k/PTEN signaling pathway thus metabolic biomarkers in addition to molecular biomarkers elucidate the challenges fronting profitable therapy of mammary cancer and an pioneering approaches that metabolomics compromises to improve recognizing drug resistance in breast carcinoma.

## Highlights

• Impact of DOX against breast cancer resistant cell lines MCF-7.
• Metabolomics analysis for deviated metabolites.
• *In vivo* induction of breast cancer via DMBA.
• DOX impact on DMBA induced breast cancer.
• Monitoring EGFR, AKT, P53, PI3K, ABCA1, PTEN *in vivo*.

## Graphical Abstract

**Figure:**
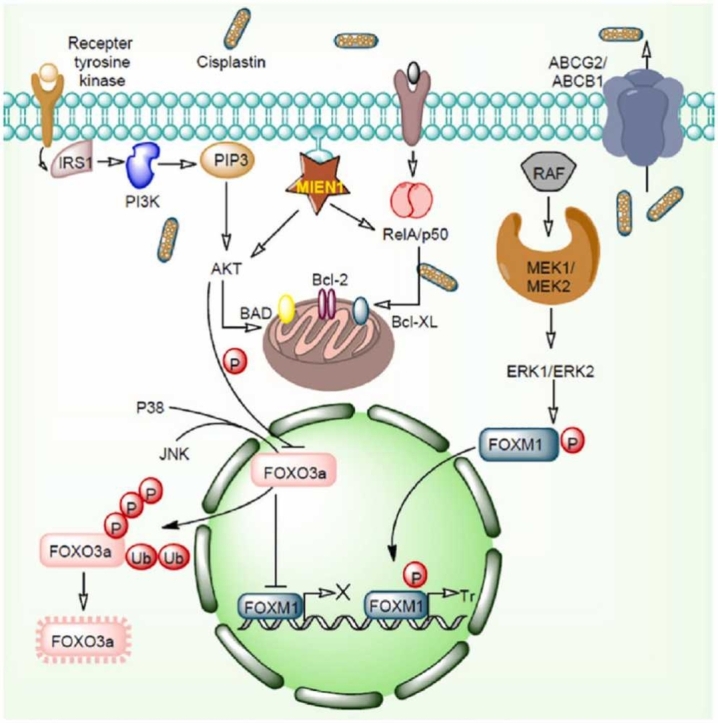

## 1 Introduction

Globally, breast cancer has the highest incidence/fatality rates it presents the second among female’s cancers and occurs due to the abnormal growth of breast cells [65]. Breast cancer initiates in milk ducts or lobules and then can spread to lymph nodes or other organs. triple-negative breast cancer (TNBC) is a common subgroup of BC accounts for nearly 15–20 % of all BC cases and conveys the worst prognosis of hormone-receptor-positive BC. Although there are various therapies available for TNBC, chemotherapeutic resistance remains a major challenge in managing BC. Chemotherapy remains the cornerstone for BC therapy and BC can be genetically resistant to chemotherapeutic treatment. Strategies for breast cancer treatment depend on the tumor's biological features. If the tumor is low grade, in situ, noninvasive, node-negative and has an estrogen receptor; hormone treatment is recommended. If the tumor is invasive high grade and/or node positive, chemotherapy is frequently administered prior to targeted therapies [51]. Understanding how cells respond to chemotherapeutic medications can lead to the development of effective cancer treatment strategy [10]. Despite notable progress in cancer treatment, the issue of therapeutic resistance as well as the harmful effects of these medications on normal tissue continues to pose important challenges. Drug resistance represents the major obstacle in breast cancer therapy. Chemotherapy resistance is a critical problem facing clinical trials and opening a novel avenue for innovate pharmaceutical drugs and could elucidate why nearly 80 % of the developed clinical programs are interrupted at every stage of clinical trials [53].

Stratifying breast cancer cells using novel metabolic biomarkers can help to differentiate between the therapeutic-response and therapeutic-resistant cancer cells and clarifies those who are expected to profit from a precise therapeutic approach [66]. In this context, the lack of actionable biomarkers represent the most significant challenge that would enable cancer therapy management to optimize drug efficacy that could reduce resistance rates and improve therapeutic efficiency [34].

Accordingly, metabolomics could offer numerous biomarkers that clarify proper approaches for cancer therapy and beneficial information regarding response and resistance drugs and can direct the early adaptation to the medical approach upon cancer development, progression of acquired resistance, and/or toxicities. Novel research revealed that metabolomics biomarkers markedly expand the outcome of clinical trial achievement rates, principally in the arena of cancer disease [13], [54]. This research aims to identify metabolic biomarkers in addition to genomic biomarkers that can represent an opportunity for additional success rates in medical, pharmaceutical investigations and bypassing drug resistance in breast cancer cells.

Breast cancer therapies include chemotherapy, surgery, radiotherapy, target and hormonal therapy. Chemotherapy resistance is the chief obstacle facing treatment of any type of cancer. Chemo-resistance allows carcinoma cells to survive drug attack and proliferate uncontrollably, contributing to violent metastatic progression. Carcinoma cells may be genetically resistant to the first-line chemotherapeutic drugs or attain resistance throughout long-term drug therapy [55].

The most successful chemotherapeutic medication for breast cancer treatment is doxorubicin (DOX), a member of the anthracycline family. Nevertheless, research has demonstrated that DOX can cause drug resistance and even tumor growth, which possess a negative impact on a patient's prognosis and survival. DOX resistance is still a noteworthy unresolved obstacle in cancer therapy, despite the investigation of numerous mechanisms. Researches have shown that signaling pathway interactions can enhance DOX resistance by promoting cell cycle progression, proliferation, and hindering apoptosis [3].

Metabolomics developing as an innovate approach in characterizing and categorizing biomarkers, that assists in estimating metabolites related to various diseases from tissues and biofluids [43]. Metabolomics approach is utilized in investigating toxicity, early disease diagnosis, nutritional studies and assessment of drug potential, as well as evaluating chemotherapy resistance acquirement [56]. Understanding the biochemical processes that took place or were taking place at the time of breast cancer diagnosis has been suggested to be possible through the analysis of a metabolite profile. Creating sensitive prognostic tools is also crucial in the field of chemo-resistance in order to identify each patient as unique and customize treatment with targeted strategies to optimize drug action [9].

There are numerous variables that contribute to chemo-resistance, including factors linked to the tumor or the drug [70]. The ATP-binding cassette (ABC) transporter superfamily has been shown to facilitate the outflow of chemotherapeutic medicines via PI3K/AKT, which contributes to the development and maintenance of multidrug resistance in tumor cells. Furthermore, it has been demonstrated that AKT phosphorylation positively correlates with breast cancer cell survival, migration, and apoptosis, all of which may lead to chemo-resistance [14]. Therefore, drug resistance can be overcomed by inhibiting PI3K/AKT, which makes cancer cells more sensitive to chemotherapeutic agents. Furthermore, it is widely known that cancer cell supported lipidomic remodeling that can alternate fatty acid transport [61]. The current study clarifies how cancer cells sustain fatty acid metabolism in their tumor microenvironment, and oncogenic signals to enhance cancer progression and tumorigenesis [17]. Previous investigations explained the correlation between ABC transporters and breast cancer and its role in reducing survival and resistance [37], [41].

Phosphatidylinositol-3-kinase catalyzes the transformation of PIP2 into PIP3 starting a signaling cascade that increases mTOR and AKT. The AKT/PI3K signaling cascade possess the ability to regulate several biological processes, including cell division, survival, and growth. Furthermore, several investigations recorded over 30 % of solid tumors have triggered mutations in PI3K gene expression [20].

The tumor suppressor PTEN mainly works in the cytoplasm as a lipid phosphatase, converting PIP3 back to PIP2 [44]. PTEN functions as an inhibitor of AKT activation by reducing PIP3 levels within the cell [49]. Human malignancies, mutation and loss of heterozygosity (LOH) frequently inactivate PTEN [24]. Recent research has demonstrated that nuclear PTEN is essential for maintaining chromosomal stability and for inducing G1 cell cycle arrest through its nuclear phosphatase activity [58]. Through the regulation of the PI3K/Akt/mTOR and PI3K/Akt/HIF1-α signaling pathways, PTEN's reno-protective impact improved apoptosis [31].

Epidermal growth factor receptor (EGFR) regulates MAPK/Akt/PI3K/mTOR signaling [29]. Cetuximab, a monoclonal antibody that is a combination of human and mouse proteins, specifically attaches to domain III of the extracellular part of the inactive form of EGFR [73]. This binding prevents ligands attachment. The antagonistic effect of PD-L1 binding on EGFR was activated inducing apoptosis in gastric cancer cells. The EGFR pathway controls the manifestation of PD-L1 in EGFR-mutant NSCLC via IL6/JAK/STAT3 signaling. PD-L1 expression in tumors may be connected to EGFR-mediated lipid metabolism reprogramming [42]. The chief target of the current research is to inspect the potential of metabolomics reprogramming via the regulation of genomic signaling pathways accountable for resistance associated with doxorubicin in breast cancer therapy.

## 2 Materials and methods

### 2.1 Wild and resistant breast cancer cell lines and culture conditions

i. Propagation of cultured cells:

Cell cultures were regularly, maintained according to the methodology of [1]. The wild; MCF-7 and doxorubicin resistant; MCF-7/adr cultured cells were separately; propagated in their growth medium (DMEM contained 10 % fetal bovine serum heat inactivated and 1 % penicillin/streptomycin). Cultures were continued separately, up to the 3rd passages to reach the normal morphological features of the growing cultures. Then after, confluent healthy cells were processed prior to measuring the differential metabolites in their cell lysates.

i. Cell extraction of the wild and resistant breast cancer cultures for the metabolomics analysis

Cells were processed for the metabolites measure according to [45]**.** Briefly, the confluent T25 flasks (about 3 ×10^6^ cells) were taken in their early passages from the incubator and media was removed. Immediately, ice cooled 5 ml PBS buffer was added to T25 flasks. The PBS addition was repeated thrice to wash off the waste and remove excess media.

3 ml ice cooled extraction solvent (Methanol: Acetonitrile: water) [2:1:1] was edited to T25 flask. 15 min freezing, the flasks were kept on ice prior to thawing the processed cells with gentle shaking to reach complete cell disruption. Cells were scraped into the extraction solvent on ice (4 C◦). Cell lysates were collected into sterile labeled eppendorf tubes. Cell suspension was centrifuged at 20 000 g and 4 C◦ for 10 min. Supernatant was then separated in GC-MS vials and dried in a speed vac using N2 for derivatization.

Metabolomics analysis [25]:

i. Derivatization steps

After drying of cell extracts by N2, 50 µl methoxyamine solutions (15 mg/ml methoxyamine hydrochloride in pyridine) were added to the dried products and incubated for 60 min at 70 ^ο^C. this work was then accomplished by editing 100 µl MSTFA with 1 % TMC and again incubate at room temperature for 60 min to complete the derivatization step [25], [26].

i. GC-Ms condition

GC-MS system at National Research Center, Cairo, Egypt using Agilent Technologies has HP-5M column (30 m × 0.25 mm internal diameter and 0.25 mm film thickness). The helium carrier was used at a flow rate of 1.0 ml/min with injection volume of 1 microliter in a split mode (1:10) and temperature program was 80 ^ο^C for 2 min; rising at 5 ^ο^C /min to 300 ^ο^C and held for 5 min. The electron ionization (EI) of Mass spectra was optimized at 70 eV with a spectral range 25–550. The solvent delay time started from 3.7.

#### 2.1.1 *In vivo* chemicals

7,12-dimethylbenz(a)-anthracene (DMBA), Doxorubicin were purchased from Sigma-Aldrich Co. (MO, USA). Specific primers for the expression of ABCA1, EGFR, p53, PI3k, AKT and PTEN genes were purchased from Thermo Fisher Scientific (MA, USA). RNA extraction kits and one-step SYBR green RT-PCR kits were obtained from Qiagen (Helden, Germany). Kits for determination of arginase tumor biomarker, lipid peroxide and total antioxidant capacity were provided from Biodiagnostic Co. (Giza, Egypt). All reagents and chemicals were of high analytical integrity.

#### 2.1.2 *In vivo* animal induction of breast cancer

Female Sprague-Dawley rats (100 g ± 15), were obtained from National Research Center, Egypt. The ideal parameters for their maintenance were 23 ± 5 ◦C, 50 ± 5 % humidity, and a 12 h/12 h light/dark cycle. Water and a regular food were also freely given to them. Animals used in this study were handled and cared appropriately, adhering to the National Research Center of Egypt's institutional animal ethics committee (permission no. 19–293). Every morning, the animals' capacity to obtain food and water was monitored in order to gauge their level of discomfort and suffering in addition to their external look. In addition, alterations in behavior and illness were also of concern.

Four groups of eight rats each were randomly divided.

Group1: Administered regular dose of saline and served as negative control.

Groups 2: Administered oral gavages of 50 mg/kg DMBA dissolved in olive oil [19]. Mammary malignancies were detected by palpating each rat twice a week. The rats received a booster dose of DMBA (25 mg/kg) after six weeks.

Group 3: For one month, rats received intraperitoneal injections of Doxorubicin low dose of 5 mg/kg post DMBA administration [75].

Group 4: For one month, rats received intraperitoneal injections of Doxorubicin high dose of 10 mg/kg post DMBA administration [75].

### 2.2 Blood and tissue sampling

To count tumors, study their morphology, and estimate the greatest diameter of tumors, hair was collected by shaving the area surrounding the tumor then, tumor diameter was measured. The largest tumor was utilized to calculate the maximum diameter, which was directly measured for single tumors (8 mm±1.5).

At the final stage of the current experiment, rats were anesthetized using the carbon dioxide procedure, followed by cervical dislocation for euthanasia. Blood samples were collected from sublingual vein, and then centrifuged at 4000 rpm for 15 mintes. The collected sera were stored at −20 ^ο^C for biochemical analysis. The rats were sacrificed via cervical dislocation, and the breast samples were extracted and cleaned with saline. Tissue was frozen at −80°C for biochemical and molecular assessment.

### 2.3 Tumor biomarker assessment

Arginase II Level was assessed spectrophotometrically using biodiagnostic kits Catalog # KA1609 (Biodiagnostic Co., Giza, Egypt), according to the manufacturer’s instructions. Arginase reacts with arginine and undergoes a series of reactions to form colored intermediate that can be detected at 570 nm [27].

### 2.4 Oxidative stress measurement

#### 2.4.1 Malondialdehyde determination

MDA Catalog #, MBS2540407 was estimated spectrophotometrically as thiobarbituric acid reactive substances post its reaction with thiobarbituric acid in acid medium and monitoring the absorbance at 540 nm [52].

#### 2.4.2 Total antioxidant capacity determination

TAC was measured by colorimetric method using biodiagnostic kits Cat # BO-TAC-200 (Biodiagnostic Co., Giza, Egypt), according to the manufacturer’s instructions. Fe3 + -TPTZ was reduced by antioxidant to Fe2 + -TPTZ that can be measured at 593 nm.

### 2.5 Quantitative RT-PCR analysis

Following the manufacturer's instructions, the RNeasy mini kit (Qiagen, CA, USA; C. No. 74104) was used to extract total RNA from breast tissue. Then one-step QuantiTect SYBR green RT-PCR Master Mix (Qiagen; C.No. 204243) was used for the quantification of the expression of ABCA1, P53, EGFR, PI3k, AKT and PTEN genes. The reaction was conducted in StratageneMx3000 P QPCR instrument (Agilent Technologies, CA, USA). Primer sequences were listed in Table 3. The temperature profile was as follows: 94 °C for 3 minutes, 94 °C for 20 seconds, 48–59 °C for 20 seconds (based on each primer's ideal annealing temperature), and 72 °C for 10 seconds. Forty PCR cycles were carried out. Using the comparative CT (2^-ΔΔCT^) approach, the relative expression of target genes was determined relative to β-actin, which served as the reference gene [2], [30].

**Table 1:** GC-MS assignments of targeted metabolites identified in wild and resistant cells after silylation.

**Peaks no.**	**Metabolites**	**RT (min.)**
T1	L-Valine, 2TMS derivative	6.871
T2	Hydrazine	7.654
T3	L-Norleucine, 2TMS derivative	8.171
T4	Silanol, trimethyl-, phosphate (3:1)	8.461
T5	L-Threonine, 2TMS derivative	8.681
T6	Glycine, 3TMS derivative	8.912
T7	Threonine, 3TMS derivative	11.002
T8	β-Alanine, 3TMS derivative	11.708
T9	L-Proline, 5-oxo−1-(trimethylsilyl)-, trimethylsilyl ester	13.869
T10	Phenylalanine, 2TMS derivative	16.13
T11	L-Glutamic acid, 3TMS derivative	16.278
T12	Xylitol, 5TMS derivative	18.552
T13	Azelaic acid, 2TMS derivative	19.774
T14	Citric acid, 4TMS derivative	20.617
T15	D-Fructose, 1,3,4,5,6-pentakis-O-(trimethylsilyl)-, O-methyloxime	21.869
T16	d-Ribose, 2,3,4,5-tetrakis-O-(trimethylsilyl)-, o-methyloxime	22.083
T17	D-Glucose, 2,3,4,5,6-pentakis-O-(trimethylsilyl)-, O-methyloxime	22.344
T18	Galactose oxime, 6TMS derivative	22.623
T19	D-Mannitol, 6TMS derivative	23.039
T20	palmitic acid, TMS ester	24.356
T21	Chizo-Inositol, 6TMS derivative	24.695
T22	Myo-Inositol, 6TMS derivative	25.704
T23	Oleic Acid, (*Z*)-, TMS derivative	27.265
T24	α-Linolenic acid, TMS derivative	27.407
T25	Stearic acid, TMS derivative	27.751
T26	2-Myristynoyl-glycinamide	54.609

**Table 2:** The common metabolites alterations according to PLS-DA VIP plot and Kruskal-Wallis test in studied groups.

**Metabolites**	**Wild MCF-7**	**Resistant MCF-7/adr**	**Adjusted p value** **(-log10"p")**
Mannitol	0.85 ± 0.01	−0.75 ± 0.01^a^	6.12**
Myoinositol	0.83 ± 0.01	−0.88 ± 0.04^a^	4.3**
Glycine	−0.078 ± 0.01	0.81 ± 0.03^a^	3.99*
α-Linolenic acid	0.38 ± 0.04	−0.38 ± 0.02^a^	3.81*
Oleic acid	0.36 ± 0.03	−0.37 ± 0.03^a^	3.64*
Stearic acid	0.31 ± 0.01	−0.35 ± 0.02^a^	3.5*

Cell metabolite values represent natural log-transformed area under the curve values relative to sum ± S.D. Significance was defined as adjusted P Values (-log 10 “P”).**:donates P ≤ 0.01,*:donates p ≤ 0.05.^a^ denotes to significant to wild MCF-7 group.

**Table 3:** Primers sequence designed for RT-PCR gene expression.

Primer Name	Primer sequence
Β- actin	5-CTTTGATGTCACGCACGATTTC−3 5-GGGCCGCTCTAGGCACCAA−3
AKT−1	5′ -CAT GAA GAG AAG ACA CTG ACC ATG GAAA−3′ 3′ -TGG ATA GAG GCT AAG TGT AGA CAC G−5′
PI3K	5′ -CCA GAC CCT CAC ACT CAG ATCA−3′ 5′ -TCC GCT TGG TGG TTT GCT A−3′
P53	5'-CAGCGTGA TGATGGTAAGGA-'3 5'-GCGTTGCTCTGATGGTGA-'3
PTEN	5′ -GGA ACT CCA ACA AGG GAG CA−3′ 5′ -TTC GGG GTC GGA AGA CCT TA−3′
EGFR	5’- AACACCCTGGTCTGGAAGTACG−3’ 5’- TCGTTGGACAGCCTTCAAGACC−3’

### 2.6 Statistical analysis

Metabolomics analysis was performed using metaboanalyst software (http://www.metaboanalyst.ca). Nonparametric (Mann −Whitney test) and Kruskal-Wallis test *via* SPSS version 17 were used for analysis of metabolites. In vivo analysis was represented as Mean ± SEM to express the data. Tukey's post-HOC test and one-way analysis of variance (ANOVA) were used for the statistical analysis. A significant threshold of p < 0.05 was established.

## 3 Results

### 3.1 Metabolic analysis

#### 3.1.1 Metabolites profiling of the wild and resistant types of the breast MCF-7 via GC-MS and data analysis in groups

Metabolite profiling using GC-MS coupled to multivariate data analyses were adopted for samples classification and identification of potential biomarkers related to resistance group.

A total of 26 intracellular metabolites in both the wild and resistant MCF-7 groups were identified. The identities retention times (RT) for individual component is presented in Table 1. GC- MS peaks were identified as endogenous metabolites, including free fatty acids, amino acids and sugars.

**Fig. 1:**
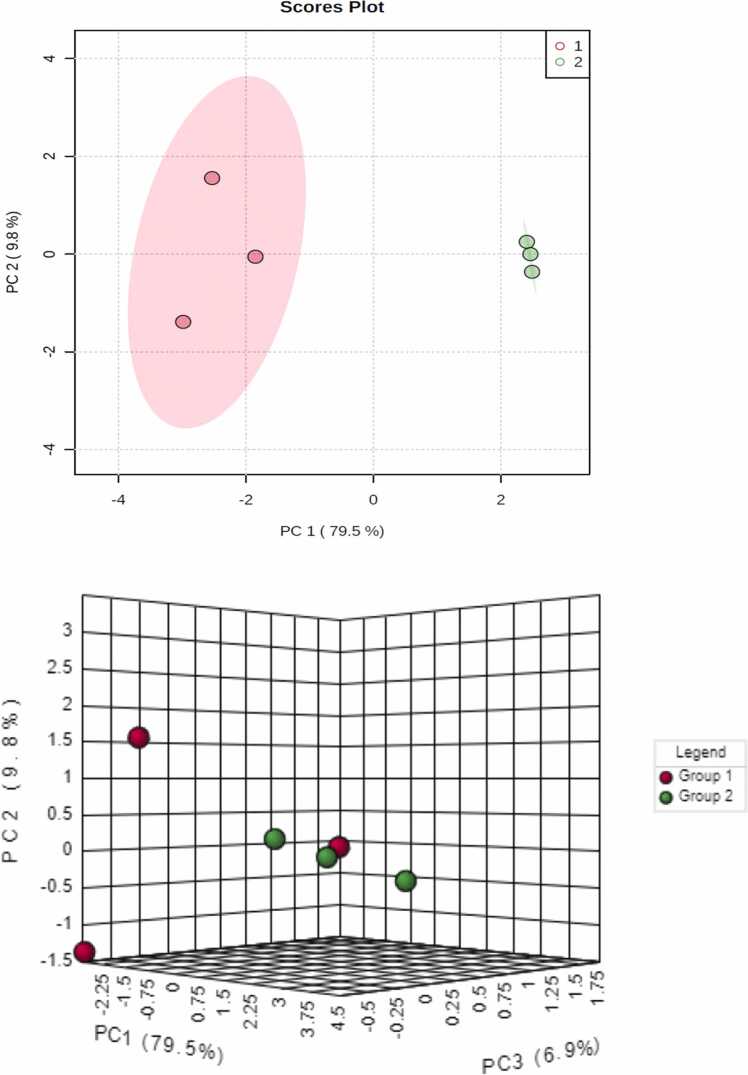
The PCA (3 D) score plots of tissue culture metabolites derived from GC-MS in both the wild MCF-7 and MCF-7/adr resistant breast cancer cell types. Group numbers 1 and 2 denote: the wild and resistant types, respectively.

Owing to the complexity of acquired data encompassing both a large number of sample size and monitored metabolites, multivariate data analyses, i.e., supervised and unsupervised models were utilized to define both similarities and differences among groups. PCA as unsupervised data analysis method was applied firstly to the GC/MS dataset with score plot (Fig. 1). Its results showed firstly clear segregation of sample groups with obvious overlap among the different replicates along PCA1. The first two components (PC1 and PC2) explained 79.5 % and 9.8 % of the total variance, respectively.

**Fig. 2:**
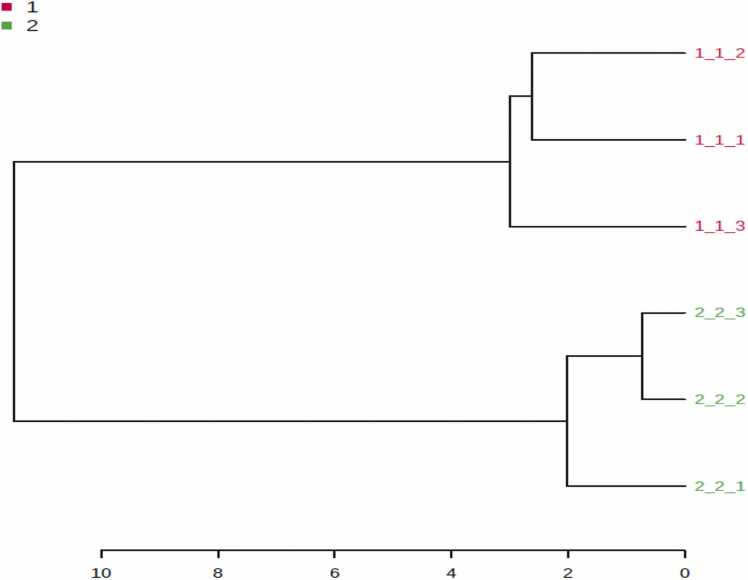
Hierarchical Clustering /Dendrograms of cell metabolites in two groups. Resistance group showed separation from the wild control group in tissue culture samples. Sample numbers are presented as follows: (1) wild MCF-7 (2) Resistant MCF-7.

For further separation between two groups and determination their arrangement according to their efficacy of these metabolites, the Hierarchical Clustering /Dendrograms model of two groups was displayed in Fig. 2.

This Dendrograms model showed that the presence of resistance group in the most distant from normal group.

**Fig. 3:**
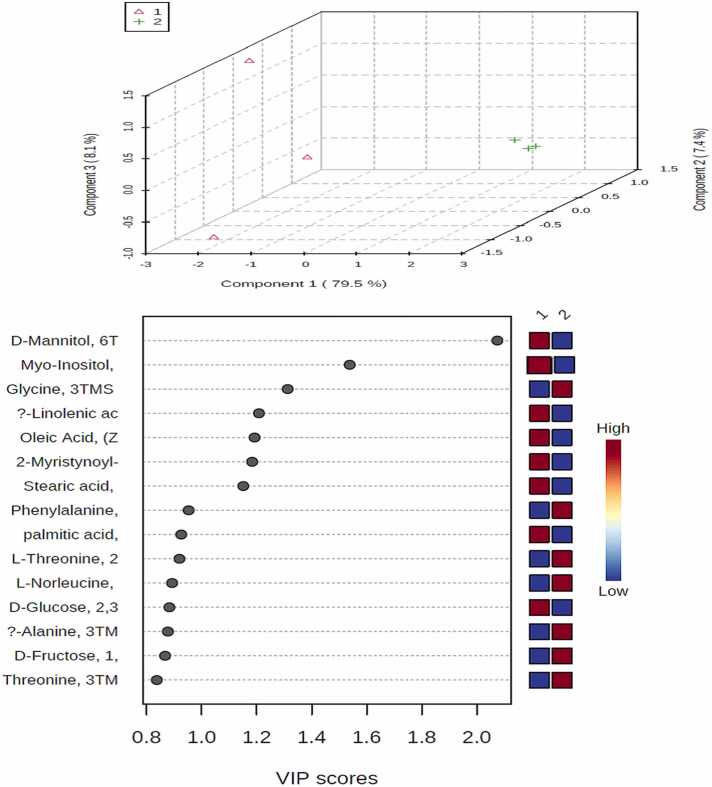
PLS-DA score plots obtained from cells GC-MS data by modeling wild MCF-7 control (1) versus MCF-7/adr resistance group (2) against each other.(A) Score plot of PC1 and PC2. (B) VIP score plot for determination of important metabolites (VIP score ≥1). Myoinositol, glycine, mannitol, α-linolenic acid, oleic acid, myristynoyl-glycinamide and stearic acid with the colored boxes on the right indicated the relative concentrations of the corresponding metabolites in each group under study.

PLS_-_DA model and its derived VIP score plot were further employed to identify metabolite markers related to resistance by modeling the parental wild control versus resistance cells (Fig. 3 A, B).

The PLS_-_DA model exhibits R^2^ value (0.99) and Q^2^ value (0.98), with wild control and resistance groups being clearly discriminated from each other. Glycine was the most elevated in resistance group, whereas Myoinositol, mannitol, α-linolenic acid, oleic acid, myristynoyl-glycinamide and stearic acid were detected at lower levels as compared to the parental wild control one.

To ensure that metabolite markers result from multivariate data analysis is significantly relevant further statistical analysis was attempted.

In agreement with the PLS_-_DA derived results, non-parametric kruskal-wallis test pointed out that six metabolites Myoinositol, glycine, mannitol, α-linolenic acid, oleic acid, and stearic acid (P < 0.05) are the most impacted metabolites between all studied groups and suggested for discrimination between wild and resistance types.

Table 2**:** showed metabolites peak AUC values from the GC MS data were log transformed into a normal distribution approximation. After log transformation, and Kruskal-Wallis test analysis using SPSS 17 was performed.

**Fig. 4:**
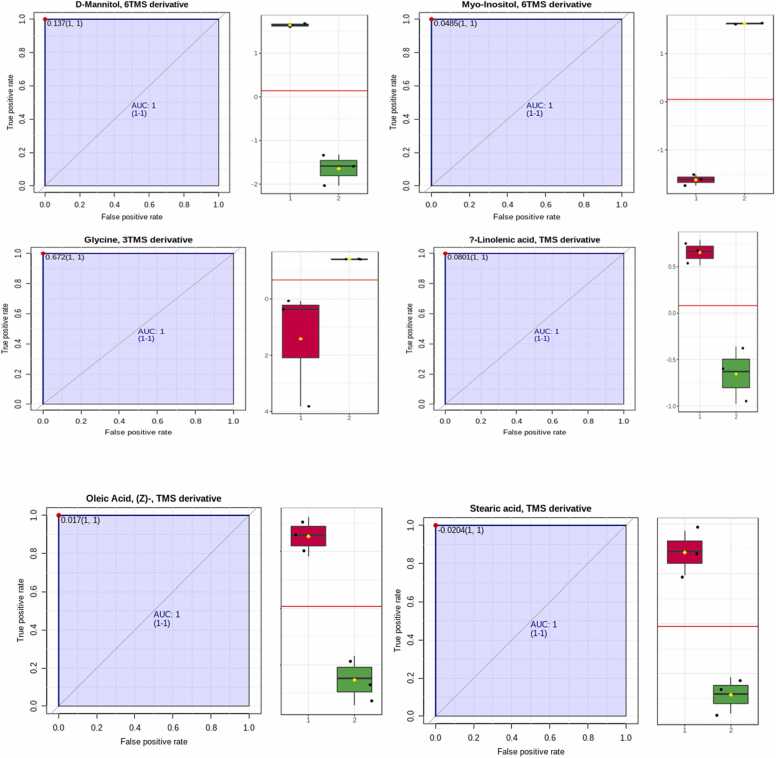
ROC curve analysis of potential cell related biomarkers to differentiate the wild and resistant groups.

Receiver Operating Characteristic (ROC) analysis was performed for those common metabolites between wild and resistance groups that showed the strongest differences between resistance cells and normal controls. As shown in Fig. 4 a, b, c, d, e, f. mannitol, myoinositol, glycine, α-linolenic acid, oleic acid and stearic acid have AUC value: 0.14, 0.5, 0.7, 0.1, 0.02, −0.02 (1, 1) respectively. As shown from these results glycine and myoinositol metabolites provided the best discriminative power in the wild and resistance groups.

### 3.2 Modulation of apoptotic biomarkers

**Fig. 5:**
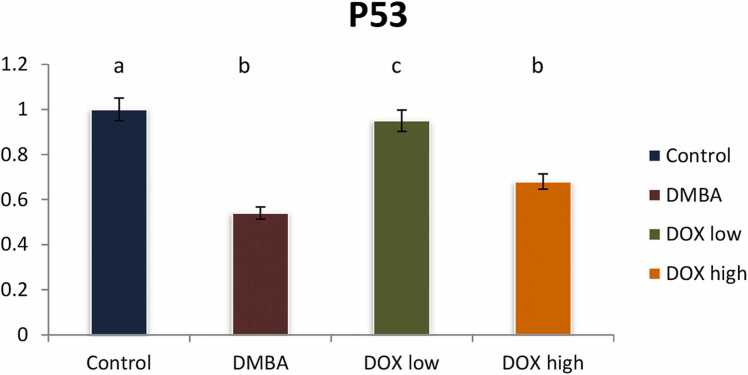
Effect of low and high dose of doxorubicin therapy on P53 gene expression after DMBA-induced breast cancer in rats. For n = 8, the data were presented as means ± SEM. It is deemed significant when p < 0.05. While groups with different letters differ significantly from one another, those with the same letter do not differ significantly from one another (If two groups carry the same letter (a) then they aren’t significantly different from one another but if they carry different letters as a, b & c then they are significantly different). β-actin was used as reference gene.

**Fig. 6:**
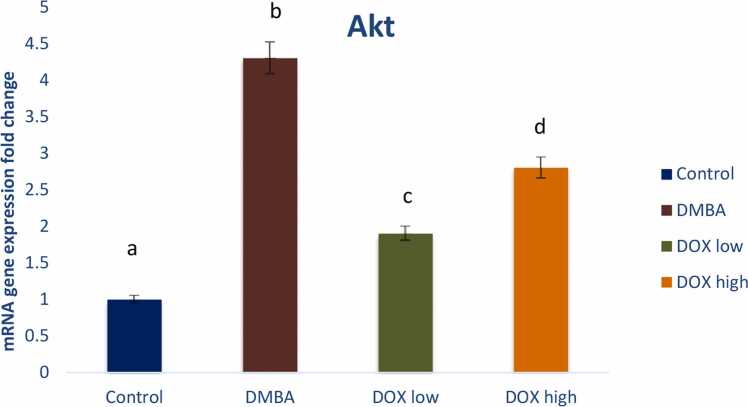
Effect of low and high dose of doxorubicin therapy on AKT gene expression after DMBA-induced breast cancer in rats. For n = 8, the data were presented as means ± SEM. It is deemed significant when p < 0.05. While groups with different letters differ significantly from one another, those with the same letter do not differ significantly from one another. (If two groups carry the same letter (a) then they aren’t significantly different from one another but if they carry different letters as a, b & c then they are significantly different). β-actin was used as reference gene.

**Fig. 7:**
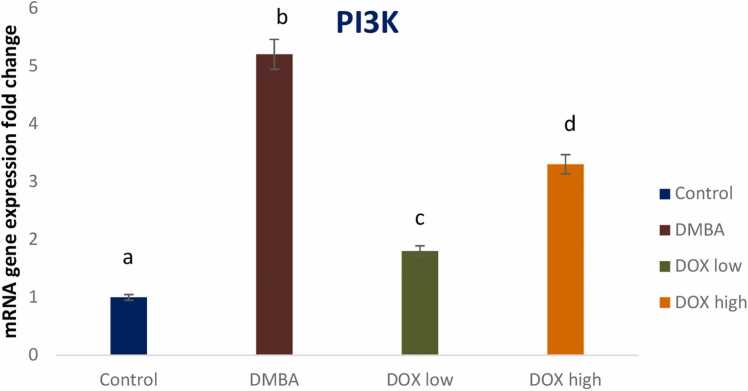
Effect of low and high dose of doxorubicin therapy on PI3K gene expression after DMBA-induced breast cancer in rats. For n = 8, the data were presented as means ± SEM. It is deemed significant when p < 0.05. While groups with different letters differ significantly from one another, those with the same letter do not differ significantly from one another. (If two groups carry the same letter (a) then they aren’t significantly different from one another but if they carry different letters as a, b & c then they are significantly different). β-actin was used as reference gene.

**Fig. 8:**
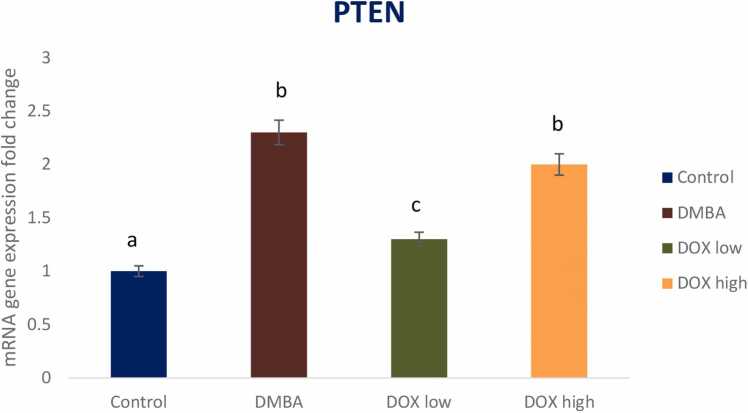
Effect of low and high dose of doxorubicin therapy on PTEN gene expression after DMBA-induced breast cancer in rats. For n = 8, the data were presented as means ± SEM. It is deemed significant when p < 0.05. While groups with different letters differ significantly from one another, those with the same letter do not differ significantly from one another. β-actin was used as reference gene.

DMBA induced breast cancer in rats declared a significant reduction in the tumor suppressor gene P53 and a significant elevation in oncogenic biomarkers of Akt, PI3K and PTEN gene expression by 2, 4.3, 5.2, and 2.3 fold changes respectively. Treatment by Doxorubicin in both low and high doses significantly modulated these signaling pathways with high dose revealing treatment resistance (Fig. 5, Fig. 6, Fig. 7, Fig. 8).

### 3.3 Expression of ABCA1 and EGFR genes

**Fig. 9:**
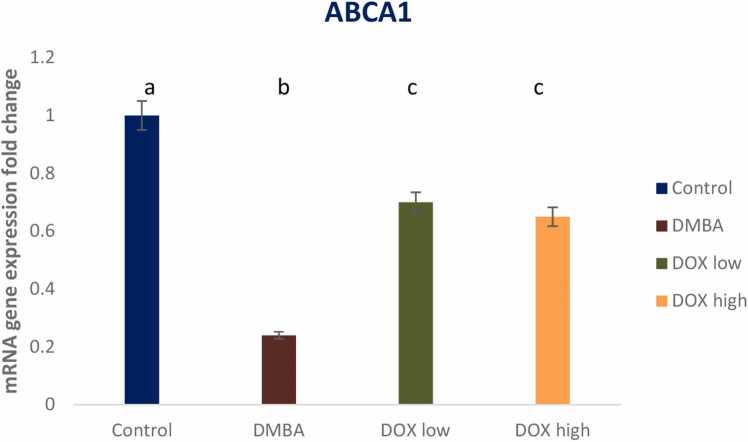
Effect of low and high dose of doxorubicin therapy on ABCA1 gene expression after DMBA-induced breast cancer in rats. For n = 8, the data were presented as means ± SEM. It is deemed significant when p < 0.05. While groups with different letters differ significantly from one another, those with the same letter do not differ significantly from one another. (If two groups carry the same letter (a) then they aren’t significantly different from one another but if they carry different letters as a, b & c then they are significantly different). β-actin was used as reference gene.

**Fig. 10:**
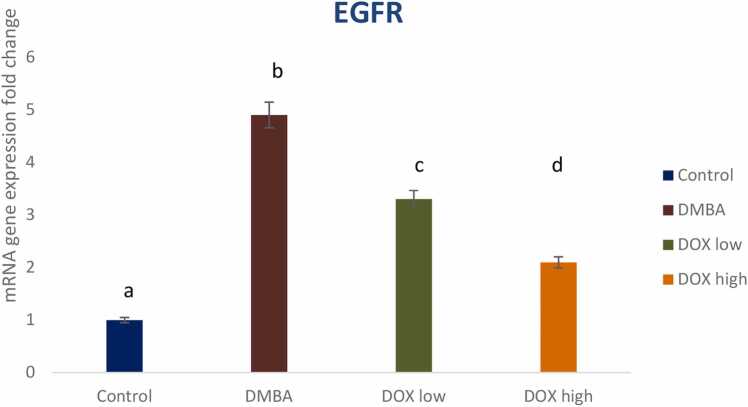
Effect of low and high dose of doxorubicin therapy on EGFR gene expression after DMBA-induced breast cancer in rats. For n = 8, the data were presented as means ± SEM. It is deemed significant when p < 0.05. While groups with different letters differ significantly from one another, those with the same letter do not differ significantly from one another. (If two groups carry the same letter (a) then they aren’t significantly different from one another but if they carry different letters as a, b & c then they are significantly different). β-actin was used as reference gene.

Breast cancer induction declared a significant reduction in the expression of ABCA1 and P53 gene expression (0.24 & 0.4 fold change) as compared with the negative control group post DMBA induced breast cancer. On the other hand, a significant up regulation in the expression of ABCA1 was noticed upon doxorubicin low and high dose treatment recording 0.7 and 0.65 fold changes, respectively with high dose revealing treatment resistance (Fig. 9). Data were expressed as means ± SD (n = 8); p < 0.05 is considered significant. On the other hand, a significant elevation in EGFR gene expression upon DMBA intoxication recording 4.9 fold changes as compared to negative control group was noticed. Meanwhile, an obvious down regulation of EGFR gene expression upon treatment by doxorubicin recording 3.3 fold change (Fig. 10).

### 3.4 Modulation of oxidative stress biomarkers

**Fig. 11:**
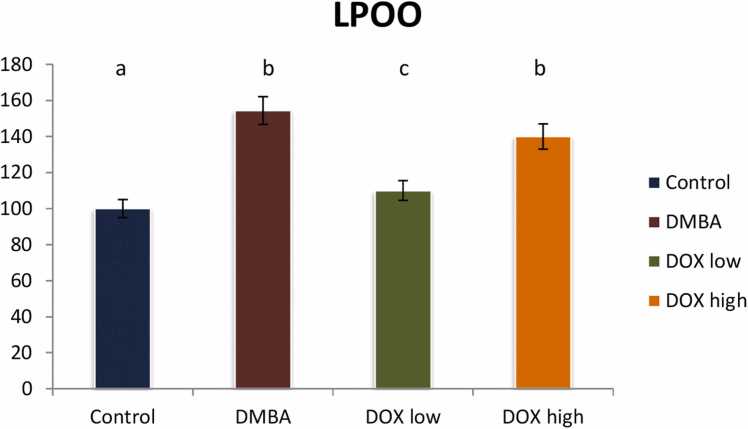
Effect of low and high dose of doxorubicin therapy on LPOO after DMBA-induced breast cancer in rats. For n = 8, the data were presented as percent change. It is deemed significant when p < 0.05. While groups with different letters differ significantly from one another, those with the same letter do not differ significantly from one another. (If two groups carry the same letter (a) then they aren’t significantly different from one another but if they carry different letters as a, b & c then they are significantly different).

Rats induced breast cancer showed a significant increment in lipid peroxide level (p < 0.05) by 154 % referring to the control group, while rats treated with doxorubicin low dose declared a significant reduction while high dose revealed treatment resistance in lipid peroxide value (p < 0.05) by 29 and 85 %, respectively as compared with positive control group. It indicates an improvement rate of 44 and 43 %, respectively as describe in Fig. 11.

**Fig. 12:**
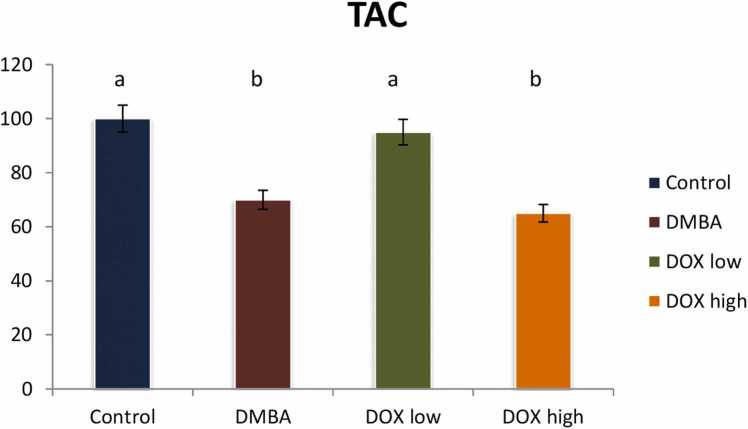
Effect of low and high dose of doxorubicin therapy on TAC after DMBA-induced breast cancer in rats. For n = 8, the data were presented as percent change. It is deemed significant when p < 0.05. While groups with different letters differ significantly from one another, those with the same letter do not differ significantly from one another. (If two groups carry the same letter (a) then they aren’t significantly different from one another but if they carry different letters as a, b & c then they are significantly different).

Furthermore, DMBA intoxicated group declared a significant reduction in total antioxidant capacity level (p < 0.05) by 58 % referring to the control group, meanwhile animals treated either with doxorubicin low or high doses showed a significant elevation in total antioxidant levels (p < 0.05) by 65 & 95 %, as compared to positive control group. It indicates an improvement rate of 40 % while high dose revealing treatment resistance as represented in Fig. 12.

### 3.5 Modulation of tumor biomarkers

**Fig. 13:**
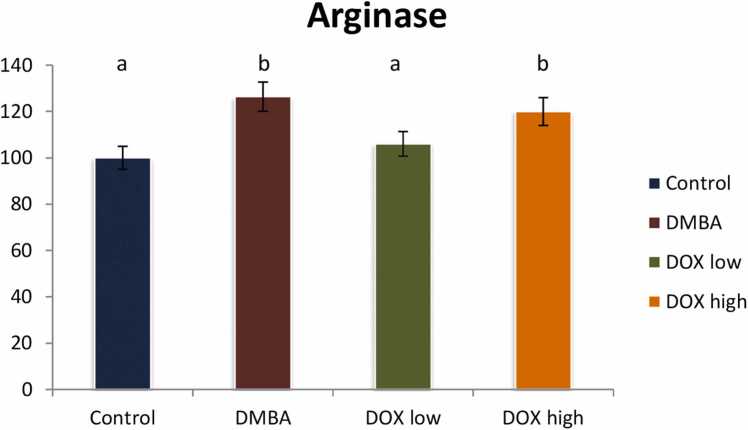
Effect of low and high dose of doxorubicin therapy on LPOO after DMBA-induced breast cancer in rats. For n = 8, the data were presented as percent change. It is deemed significant when p < 0.05. While groups with different letters differ significantly from one another, those with the same letter do not differ significantly from one another. (If two groups carry the same letter (a) then they aren’t significantly different from one another but if they carry different letters as a, b & c then they are significantly different).

DMBA intoxicated animals illustrated a significant increase in Arginase level (p < 0.05) by 126 % referring to the control group, while rats treated either with doxorubicin low or high doses declared a significant reduction in Arginase level (p < 0.05) by 16 & 5 % as compared to positive control group. It indicates an improvement rate of 20 % and high dose revealing treatment resistance as describe in Fig. 13.

## 4 Discussion

Metabolomics is a hallmark widely applied in population-based statistics for the documentation of novel etiologic hypotheses associated with breast cancer (BC) progress and drug resistance. Regardless of its achievement in applications in epidemiological statistics, larger sample size with detailed data on breast cancer kinds, and repetitive biologic samples collected over time are required to develop comparison among studies results and results validation improvement, allowing potential clinical translation of the metabolic outcomes [46].

Irrespective to biochemical subtypes, drug resistance in most types of BC are still unexplained clinical problematic. Although majority of BC rely on endocrine treatment, about 30 %–60 % of these cancers exhibit acquired or de novo resistance [65]. Even though numerous researches utilizing genomic technologies recently provided a glimpse into the complicated genetic makeup of BC subgroups and the interlink between chemotherapeutic resistance, survival or metastatic progress. Light was shaded on the endoplasmic reticulum-controlled signaling that could contribute to anti-estrogen resistant phenotype, documentation on specific biomarkers of anti-estrogen responsiveness still indefinable [33]. In HER2-positive BC; trastuzumab therapy beside standard chemotherapy significantly improved survival in the past decade; nevertheless, resistance stills a life-threatening setback. Crosstalk among PI3K/AKT signaling cascade is amongst several causes contributing to trastuzumab resistance [74]. Chief regulators of cellular metabolomics as P53, PI3K m-TOR and MYC were highly mutated in numerous subtypes of BC. Up regulation in these pro-survival genes permits BC cells to perform quick recovery from glucose/ATP or amino acid synthesis disturbance induced via chemotherapy. Metabolomics analysis revealed elevated aerobic glycolysis rate relatively due to more energy-efficient mitochondrial oxidative phosphorylation, highlighting more energy needs for cancer cells growth. With abnormally elevated cell proliferation rates, the rate of aerobic glycolysis, glutaminolysis or fatty acid synthesis is also abnormally promoted in BC cells to keep up both energy and biomass demands. For the intervention of novel therapeutic approaches for cancer elimination, lipidomic and genomic analysis of chemo-resistant cancer cells can be used to identify and discover potential targets. However, very few studies have investigated the metabolic profile of chemo-resistant breast cancer cells [12]. This work, generated and characterized a wild type; MCF-7 and doxorubicin resistant; MCF-7/adr cultured cell lines and investigated its metabolites; integrated analysis of the omics data pinpointed several significant alterations compared to the parental cell line, suggesting that they may be exploited to target the resistant cells. Metabolomics profiling using GC- MS a wide-spread technique in addition to molecular profiling to resistance, apoptotic and cell survival genes were performed in breast cancer resistance cell lines [48].

In our study, we noticed six vital metabolites were involved discriminating between wild and resistance BC cells. These metabolites were mannitol, α-linolenic acid, oleic acid, myoinositol and stearic acid which were decreased in resistance cells but glycine was elevated. The most promising metabolites were myoinositol and glycine.

Glycine biosynthetic pathway was highly correlated with fast proliferating breast cancer cells. [28] suggested that glycine consumption is required for cancer cell proliferation, and is associated with worse prognosis in breast cancer patients. This finding also suggested a potential cancer biomarker and therapeutic response tracking.

Numerous metabolites exert a wide range of physiologically and pathologically vital activities as inositol (myo-Ins). Inositol can be investigated in several metabolic diseases as neurological and polycystic ovary syndrome [7].

In this study the myoinositol was decreased in cancer cells compared to their parental wild one. Deregulation of inositol metabolism has been associated with cancer, and myoinositol has been found to exert multiple anti-cancer effects, including pro-apoptotic and anti-proliferative ones in various cancer types. In a colorectal adenocarcinoma cell line, the inositol 3-phosphate synthase (ISYNA1) enzyme, which is essential for myoinositol biosynthesis, was found to be a direct target of P53 [39]. It was suggested that P53 suppressed tumorigenesis by inducing myo-inositol biosynthesis. ISYNA1 knock-down resulted in resistance to doxorubicin treatment, proposing a role for myo-inositol biosynthesis in P53-mediated growth suppression [35]. However, there are some reports arguing against the involvement of P53 in the anti-cancer activity of myo-inositol. This was confirmed by the current *in vivo* study that revealed P53 reduction post DMBA administration meanwhile; P53 was elevated post doxorubicin low dose administration and reduced post the high dose administration. This was interpreted to that P53 activation, suppresses many genes involved in the cell division cycle. For instance, Kinase family member’s phosphatidylinositol 3-kinases/AKT serine/threonine kinase-1 (PI3K/AKT) and cyclins were hindered upon P53 elevation. Furthermore, P53 overexpression triggers apoptosis of breast cancer cell lines via stimulating NOXA, Bcl-2-associated X protein (BAX) and P53-upregulated modulator of apoptosis (PUMA). P53 was conjugated to the anti-apoptotic marker Bcl-2 that exists in the mitochondria thus enhance endogenous apoptosis via Apaf-1/cyt-c signaling pathway. Mutation in P53 the tumor suppressor gene was prominent in several cancer types, comprising breast cancer. Other groups have shown that myo-inositol inhibits the epithelial-to-mesenchymal program [35] and inflammatory factors in breast cancer cells and that it induces the regression of different types of cancer. Notably, in a prospective, randomized study, inositol hexakisphosphate and myo-inositol improved the responsiveness to adjuvant therapy in breast cancer patients and markedly increased their quality of life. Therefore, mounting evidence suggests that myo-inositol exerts pleiotropic anti-cancer effects and, according to our data, its reduced levels could be a marker of paclitaxel resistance in triple negative breast cancer (TNBC). It is tempting to speculate that this metabolite mediates chemo-sensitivity, however, further mechanistic studies are needed to unequivocally establish such a property for myo-inositol and uncover the underlying cellular pathways [40].

Numerous results reported that, the most important nutrients expended by cancer cells including those resistant to anticancer drugs are glucose and glutamine [57]. Many resistant cells contribute to increase in glucose and/or glutamine up take followed by mitochondrial oxidation via metabolic genes deregulation. Many lipid metabolisms are reported in deregulation of cancer [62].

α-linolenic acid, oleic acid and stearic acid were detected in lower levels in resistance group due to the over consumption as compared to the wild control one. It was elucidated in several studies that lipids and lipid droplets are accumulated in many types of cancer including breast and lung cancer. Numerous complex processes were involved in deregulation of lipid metabolism in BC cells. One of the major features of cancer cells is the de novo-lipid synthesis, suggesting that its inhibition might yield an acceptable therapeutic index.

The lipid biosynthesis in cancer cells is mainly due to supplies of carbon unit from glucose for lipogenesis. By using glycolytic pathway alternative carbon sources can be used. During hypoxia, glutamine can replace glucose and form α-ketoglutarate (αKG) which will make reductive carboxylation via isocitrate dehydrogenase 1 (IDH1) to produce citrate and thus can significantly contribute to lipid biosynthesis in cancer cells. Overexpression of acetyl-CoA synthetases (ACSS) was detected in human breast cancer through the parallel pathway for acetyl-coenzyme A (CoA) production for lipogenesis, independent of citrate conversion to acetyl-CoA by acetyl - CoA synthetases (ACSS) [67].

Human breast cancers overexpress Acetyl-CoA synthetase-2 (ACSS2), and are thus critically dependent on acetate for lipid synthesis [63]. These observations underline the intricate relationship between glycolysis, glutaminolysis, acetate metabolism and lipogenesis in cancer. High level of de novo FA synthesis results in (i) overproduction of neutral lipids such as triacylglycerols stored in LD that accumulate in cancer to provide a reserve of energy; (ii) production of phospholipids is used to build cancer cell membranes to satisfy the increased demand for cancer proliferation. Moreover, phospholipids also act as lipid messengers and intracellular signaling molecules in cancer [21]. Among lipogenic enzymes, ATP citrate lyase (ACLY), the rate-limiting enzymes acetyl-CoA carboxylase (ACC) and fatty acid synthase (FAS), are the most expressed enzymes in many cancer types [47]. Particularly, FAS was identified as the lipogenesis key enzyme, and it’s up regulation has been correlated with a bad prognosis in many types of cancer. Human cancers, including breast, colon and prostate cancer, have high expression and activation levels of fatty FAS, increasing the triacylglyceride (TG) synthesis stored LDs. Thus, FAS targeting in cancer is of growing interest [6]. Orlistat, a drug used for obesity treatment, was shown to target FAS by inhibiting the FAS thioesterase function leading to anti-tumor activity. Besides, up regulation of the mevalonate biosynthesis pathway has been observed in many cancer types. This leads to cholesterol overproduction coming from the conversion of acetyl-CoA via the 3-hydroxy-3-methylglutaryl-CoA (HMG-CoA) reductase. Cholesterol content in cancer cell membranes and cholesterol rates were found to be aberrantly high in prostate cancer, leading to promoted cancer growth [18]. Many enzymes within the fatty-acid and cholesterol-biosynthesis pathways were up regulated in cancer by the sterol regulatory element-binding proteins (SREBP) transcription factors activated by the oncogenic PI3K/Akt/mTORC1 (mammalian target of rapamycin) signaling pathway or cell cycle regulators [31]. This finding was confirmed by *in vivo* current study, that revealed elevated oncogenic biomarkers including Akt, PI3K, and PTEN gene expression post DMBA induced breast cancer. Meanwhile, Doxorubicin treatment in low dose markedly ameliorated the oncogenic signaling pathways on the other hand; DOX high dose showed resistance and elevated these oncogenic biomarkers again. Recently, a novel target for overcoming medication resistance has been identified as the PI3K/AKT/mTOR pathway [36], [71]. Guerrero- Zotano et al. [16] found a strong correlation between the deregulation of this system and the advancement of tumors in breast cancer as well as resistance to conventional therapy. One of the pathways that are most frequently activated in a variety of cancer types is the PI3K/AKT/mTOR pathway [11], [5]. Several pharmaceuticals that target the PI3K/AKT/mTOR pathway are undergoing clinical trials. In this study, we provide an efficient drug development plan and a summary of the current understanding of the PI3K/AKT/mTOR pathway in relation to drug resistance in breast cancer [32].

Secondly, apart from lipogenesis, it was observed that FAs, either from extracellular sources or mobilized from internal lipid stores, can be oxidized in cancer cell mitochondria. Under these conditions, lipids are used as catalytic fuels, a process called fatty acid oxidation (FAO) or lipolysis, to provide energy for cancer cells via ATP production. In some cancers, not dependent on glycolysis like B cell lymphoma, mitochondrial FAO represents the predominant pathway for energy production [69].

It is now well-established that lipid metabolism changes are associated with resistance to conventional chemotherapies and targeted therapies in several cancers. Lipid metabolic reprogramming of resistant cancer cells includes both changes in de novo lipogenic synthesis and/or lipolytic pathway. With some exceptions [72], cancer cell resistance appears to be related to the up regulation of lipogenic or lipolytic enzyme expression. Firstly, changes in lipid metabolism of resistant cells are to some extent treatment-specific. Resistance to tyrosine kinase inhibitors (TKIs) is associated with up regulation of de novo lipogenesis. LD accumulation, resulting from up regulation of lipogenesis, is higher in epidermal growth-factor (EGF)/TKI resistant cell lines, with aberrant activation of EGF receptor (EGFR) signaling pathway, than in cell lines with sensitive EGFR mutations [22]. This is consistent with the crucial role of the receptor tyrosine kinase signaling pathway sustaining up regulation of sterol regulatory element-binding protein (SREBP)-driven de novo lipogenesis. EGFR-TKI-resistant non-small-cell lung carcinoma (NSCLC) cell lines are characterized by an accumulation of LD and overexpression of Stearoyl-CoA Desaturase 1 (SCD-1), a key enzyme converting saturated fatty acids into unsaturated fatty acids [64]. Conversely, resistance to mitogen-activated protein kinase (MAPK) pathway inhibitors has generally been associated with increased FAO in BRAF mutated melanoma cells and this agreement with our finding which decreases these free fatty acids due to the increase of fatty acids oxidation [15]. The *in vivo* current study revealed a significant overexpression in EGFR gene expression in addition to an obvious reduction in ABCA1 gene expression upon DMBA induced breast cancer in rats. Meanwhile, Doxorubicin treatment in low dose markedly ameliorated EGFR and elevated ABCA1 gene expression on the other hand, DOX high dose showed resistance and elevated EGFR and reduced ABCA1 gene expression. Due to its involvement in the development of cancer and its potential as a prognostic factor, EGFR is the receptor tyrosine kinase that has been studied in various studies [4]. Chemotherapeutic drugs that damage DNA, like DOX, can cause severe side effects. For instance, it has been demonstrated that DOX therapy causes cardiomyocyte senescence and oxidative stress, which can lead to potentially fatal cardiomyopathy [8]. Many studies have concentrated on DOX combination therapy in an effort to prevent such occurrences. The objective is to identify synergistic medication combinations that would enable DOX to be administered in lower dosages, reducing toxicity, while still producing the same or improved efficacies. Several molecular processes, such as reduced drug accumulation, metabolic detoxification, anti-apoptosis, autophagy, and others, are thought to be responsible for the biological reasons of drug resistance [68]. As previously recorded, PI3K/AKT induces resistance to chemotherapy by means of proteins linked to multidrug resistance and anti-apoptotic properties [76]. The ATP-binding cassette (ABC) transporter superfamily has been shown to facilitate the outflow of chemotherapeutic medicines via PI3K/AKT, which contributes to the development and maintenance of multidrug resistance in tumor cells [77]. Furthermore, it has been demonstrated that AKT phosphorylation positively correlates with breast cancer cell survival, migration, and apoptosis, all of which may lead to chemo-resistance [14]. Therefore, drug resistance can be overcome by inhibiting PI3K/AKT, which will make cancer cells more sensitive to chemotherapeutic agents. Furthermore, it is widely known that cancer cell supported lipidomic remodeling that can alternate fatty acid transport [61]. The current study clarifies how cancer cells sustain fatty acid metabolism in their tumor microenvironment, and oncogenic signals to enhance cancer progression and tumor-genesis. ABCA1 was the primary discovered human ABC transporter and was accountable for modulating drug permeability. ABCA1 is highly expressed in breast, BBB and kidney. ABCA1 exports positive or neutral charged hydrophobic compounds and xenobiotics outside the cell, thus protecting cell versus cytotoxicity. In spite of its defensive role in cell protection, ABCA1 mRNA overexpression in breast cancer specimens signifies a poor chemotherapeutic response, leading to low survival rates. ABCA1 can efflux chemotherapeutic agents and decrease intracellular drug levels, which is the major contributor to chemotherapeutic-resistance [77].

Since DMBA changed the standard biochemical parameters of the animal body, altered antioxidant enzyme activities, increased reactive oxygen species (ROS) & lipid peroxidation which resulted in damaging the cellular structure and membrane of organelles including: DNA, lipids & proteins as represented by Rojas-Armas et al. [60]. This result agreed with Kinoshita et al. [38] & Rojas-Armas et al. [60]. Doxorubicin resistance is a major problem in cancer therapy causing poor patient prognosis and survival. Several previous investigations have illustrated that signaling pathways interaction can enhance drug resistance via progression of cell cycle, proliferation induction, oxidative stress and apoptosis prevention [59]. The main target of the current study was to investigate the impact of doxorubicin on oncogenic signaling pathways as well as signaling pathways responsible for the activation of drug resistance [23].

The pathogenesis of various diseases is influenced by oxidative stress. Our study revealed that DMBA elevated MDA, Arginase and TAC levels when compared to control and Doxorubicin treated groups. This alteration is due to the increased formation of ROS, which caused damage to a variety of biomolecules and had a variety of molecular and cellular effects, including cytotoxicity and mutagenicity, which can lead to cancer onset and progression. This result came in accordance with Nguedia et al. [50].

## 5 Conclusion

The data of the current research through application of metabolomics and genomic technologies in identifying metabolic and molecular biomarkers for drug responsiveness provides non-invasive accurate techniques to characterize breast cancer resistance. Identification of metabolic biomarkers explores the target metabolic pathways that promote cell survival and drug resistance. Efficient analysis tools for large volumes of data from breast cancer cell models and metabolomics data to translate the high-throughput information to drug responsiveness can help to accelerate the translation of new findings in the laboratory to the clinical diagnostic and prognostic lab.

## Funding

The current work is financially supported by the National Research center, internal projects grant number 12020102.

## Author statement

All authors revised the manuscript and agree for publication

## CRediT authorship contribution statement

**Rehab M Abdel-Megeed:** Writing – review & editing, Methodology, Investigation. **Abdel Hamid Z Abdel Hamid:** Methodology. **Maha M. Soltan:** Investigation. **Heba A Hassan:** Methodology. **Naglaa M Ammar:** Investigation. **Nahla N. Kamal:** Investigation. **Noha S Hussein:** Formal analysis, Data curation. **Gamal Eldein Fathy Abd-Ellatef:** Methodology. **Mai osman Kadry:** Methodology, Investigation, Formal analysis.

## Declaration of Competing Interest

The authors declare that they have no known competing financial interests or personal relationships that could have appeared to influence the work reported in this paper.

## Data Availability

No data was used for the research described in the article.
